# Supplementary material for: Real-World Outcomes of Immunotherapy in Second- or Later-Line Non-Small Cell Lung Cancer with Actionable Genetic Alterations
Source: Cancers (Basel). 2023 Nov 16;15(22):5450. doi: 10.3390/cancers15225450 (PMC10670305; doi:10.3390/cancers15225450)
Supplement: Supplementary file 1 [file cancers-15-05450-s001.zip › Supplementary_Tables and Figures_Track change.pdf]

Supplementary contents

Table S1. Clinical Characteristics of Patients Receiving ICI Monotherapy as Second-Line or Later-Line Treatment

Table S2. Univariate analysis of the predictive factors of PFS in patients with AGA mutations

Table S3. Multivariate Analyses of the Predictive Factors of PFS in Patients With AGA Mutations (*EGFR*, *KRAS*, *HER2*, and *MET subgroups*)

Table S4. PFS analysis of PD-L1 expression in patients with AGA mutations (*EGFR*, *KRAS*, *HER2*, and *MET subgroups*).

Figure S1. Patient flow diagram

Figure S2. *KRAS* subtypes (A) Frequency rate of *KRAS* subtypes (B) mPFS per *KRAS* subtype.

PFS, progression-free survival

Table S1. Clinical characteristics of patients receiving ICI monotherapy as second-line or later-line treatment.

**AGA type n (%)**		**AGA n = 324**	**Wild Type n = 602**	***p***
Demographic data				
Age	Median age (95% range) (years)	63.1 (39.3-81.7)	66.9 (44.8-82.4)	
	<65	183 (56.5)	234 (38.9)	*<0.001*
	≥65	141 (43.5)	368 (61.1)	
Sex (n)	Male	172 (53.1)	527 (87.5)	*<0.001*
	Female	152 (46.9)	75 (12.5)	
Smoking	Never	174 (53.7)	105 (17.4)	*<0.001*
	Ex or Current	150 (46.3)	497 (82.6)	
ECOG	0-1	315 (97.2)	584 (97.0)	1.0
	≥2	9 (2.8)	18 (3.0)	
Histology	Non-squamous	290 (89.5)	313 (52.0)	*<0.001*
	Others	34 (10.5)	288 (47.8)	
**Line of Therapy**	**2nd line**	**153 (47.2)**	**524 (87.0)**	***<0.001***
	**Later line**	**171 (52.8)**	**78 (13.0)**	
Concomitant disease & therapy				
HTN	No	193 (59.6)	345 (57.3)	0.8
	Yes	129 (39.8)	253 (42.0)	
	NI	2 (0.6)	7 (1.2)	
DM	No	244 (75.3)	443 (73.6)	0.6
	Yes	79 (24.4)	154 (25.6)	
	NI	1 (0.3)	5 (0.8)	
RT	No	280 (86.4)	453 (75.2)	*<0.001*
	Yes	44 (13.6)	149 (24.8)	
Steroid †	No	248 (76.5)	479 (79.6)	0.3
	Yes	76 (23.5)	123 (20.4)	
Antibiotics$	No	273 (84.3)	495 (82.2)	0.5
	Yes	51 (15.7)	107 (17.8)	

Note: Values are shown as number (%) unless indicated otherwise.

* including ccrt; † steroid use to treat irAE during ICI therapy; $ antibiotic use during ICI therapy; ICI, immune checkpoint inhibitor; AGA, actionable genetic alteration; ECOG, Eastern Cooperative Oncology Group performance status; HTN, hypertension; DM, diabetes mellitus; RT, radiotherapy.

Table S2. Univariate analysis of the predictive factors of PFS in patients with AGA mutations.

Biomarkers	AGA		EGFR		KRAS		HER2		MET		ALK		BRAF		ROS1		RET	
	HR (95% CI)	*p*	HR (95% CI)	*p*	HR (95% CI)	*p*	HR (95% CI)	*p*	HR (95% CI)	*p*	HR (95% CI)	*p*	HR (95% CI)	*p*	HR (95% CI)	*p*	HR (95% CI)	*p*
Age (≥65, vs <65)	0.76 (0.60-0.96)	*0.02*	0.72 (0.50-1.01)	0.1	0.71 (0.43-1.20)	0.21	0.90 (0.43-1.87)	0.78	0.65 (0.28-1.51)	0.32	0.15 (0.02-1.22)	0.08	4.11 (.66-25.79)	0.13	NC		0.76 (0.14-4.20)	0.75
Gender (Female, vs Male)	1.20 (0.95-1.51)	0.13	0.94 (0.67-1.32)	0.7	1.40 (0.84-2.35)	0.20	1.19 (0.55-2.59)	0.66	1.57 (0.64-3.88)	0.98	0.73 (0.18-3.05)	0.67	1.26 (0.28-5.73)	0.76	1.25 (0.24-6.63)	0.79	NC	
Smoking (yes, vs no)	0.84 (0.66-1.06)	0.14	0.96 (0.68-1.36)	0.8	0.65 (0.39-1.09)	0.10	1.07 (0.49-2.35)	0.86	0.84 (0.36-1.94)	0.68	0.92 (0.25-3.48)	0.91	1.73 (0.38-7.79)	0.48	0.70 (0.08-6.02)	0.75	NC	
ECOG (>=2, vs 0-1)	1.58 (0.81-3.06)	0.18	2.14 (0.53-8.70)	0.3	2.54 (1.00-6.44)	0.05	1.48 (0.20-11.03)	0.70	0.85 (0.11-6.34)	0.87	NC		NC		NC		NC	
Histology (non-squamous vs. others)	1.22 (0.83-1.79)	0.32	2.05 (0.99-4.23)	0.05	0.85 (0.36-1.98)	0.71	0.76 (0.32-1.81)	0.54	1.36 (0.46-4.03)	0.58	1.85 (0.23-15.06)	0.57	0.85 (0.36-1.98)	0.71	NC		0.75 (0.08-6.73)	0.79
HTN (yes, vs no)	1.00 (0.98-1.01)	0.89	1.00 (0.98-1.02)	0.8	0.65 (0.38-1.11)	0.11	1.46 (0.66-3.20)	0.35	1.00 (0.98-1.02)	0.81	0.51 (0.11-2.46)	0.40	2.98 (0.54-16.39)	0.21	0.90 (0.22-3.66)	0.88	0.89 (0.18-4.47)	0.89
DM (yes, vs no)	1.00 (0.98-1.02)	0.72	1.06 (0.71-1.59)	0.8	1.07 (0.58-2.00)	0.83	1.76 (0.82-3.75)	0.15	1.00 (0.98-1.02)	0.81	2.46 (0.55-11.02)	0.24	1.57 (0.35-7.11)	0.56	NC		NC	
RT (yes, vs no)	1.38 (1.00-1.91)	0.05	0.87 (0.53-1.44)	0.6	1.73 (0.82-3.66)	0.15	1.45 (0.66-3.21)	0.35	NC		3.73 (0.66-20.89)	0.14	NC		0.34 (0.04-2.87)	0.32	1.34 (0.15-12.1)	0.79
Steroid † (yes, vs no)	0.62 (0.46-0.83)	*0.001*	0.73 (0.49-1.09)	0.1	0.34 (0.17-0.69)	*0.003*	0.38 (0.15-0.95)	*0.04*	0.31 (0.07-1.30)	0.11	1.51 (0.40-5.65)	0.54	NC		1.17 (0.22-6.05)	0.86	1.34 (0.15-12.1)	0.79
antibiotics$ (yes, vs no)	0.68 (0.49-0.94)	*0.02*	0.68 (0.43-1.08)	0.1	0.51 (0.26-1.01)	0.054	0.74 (0.18-3.17)	0.70	0.60 (0.14-2.57)	0.49	1.39 (0.37-5.24)	0.63	NC		NC		1.34 (0.15-12.1)	0.79
PD-L1 cutoff 1% (High, vs Low)	0.62 (0.48-0.80)	*0.0002*	NC	0.2	0.38 (0.22-0.65)	*<0.001*	0.55 (0.25-1.24)	0.15	0.54 (0.22-1.34)	0.19	3.22 (0.39-26.33)	0.28	0.24 (0.04-1.53)	0.13	1.44 (0.28-7.32)	0.66	NC	
TMB cutoff 10 (High, vs Low)	0.91 (0.54-1.52)	0.71	0.73 (0.28-1.90)	0.5	0.52 (0.17-1.59)	0.25	0.45 (0.14-1.44)	0.18	NC		NC		NC		NC		NC	
TP53 (mutation, vs wild type)	0.95 (0.69-1.29)	0.72	0.66 (0.33-1.31)	0.2	0.75 (0.44-1.25)	0.27	0.67 (0.30-1.49)	0.33	2.50 (0.68-9.12)	0.17	NC		1.87 (0.31-11.34)	0.49	NC		0.69 (0.11-4.17)	0.68
STK11 (mutation, vs wild type)	1.66 (1.00-2.45)	*0.05*	1.54 (0.37-6.43)	0.6	3.00 (1.53-5.90)	*0.001*	NC		1.29 (0.17-9.98)	0.81	NC		1.78 (0.18-17.23)	0.62	NC		NC	
KEAP1 (mutation, vs wild type)	1.40 (0.76-2.58)	0.28	5.47 (0.71-42.00)	0.1	1.85 (0.79-4.33)	0.15	0.66 (0.19-2.21)	0.50	5.15 (0.62-2.88)	0.13	NC		NC		NC		NC	

† steroid use to treat irAE during ICI therapy; $ antibiotic use during ICI therapy; NC, not calculable; PFS, progression-free survival; HR, hazard ratio; CI, confidence interval; ECOG, Eastern Cooperative Oncology Group performance status; HTN, hypertension; DM, diabetes mellitus; RT, Radio therapy; TMB, tumor mutational burden.

Table S3. Multivariate analyses of the predictive factors of PFS in patients with AGA mutations (*EGFR*, *KRAS*, *HER2*, and *MET subgroups*).

**Biomarkers**	**AGA**		**KRAS**		**EGFR**		**HER2**		**MET**	
	HR (95%CI)	***p***	***HR (95%CI)***	***p***	***HR (95%CI)***	***p***	***HR (95%CI)***	***p***	***HR (95%CI)***	***p***
Smoking (yes vs no)	1.23 (0.71-2.14)	0.46	0.83 (0.27-2.57)	0.74	NC	NC	0.79 (0.16-4.0)	0.78	0.84 (0.19-3.75)	0.82
Steroid† (yes vs no)	0.46 (0.23-0.92)	*0.03*	0.59 (0.14-2.47)	0.47	2.82 (0.68-11.8)	0.16	0.46 (0.07-3.0)	0.42	0.20 (0.02-1.81)	0.15
Antibiotics$ (yes vs no)	0.77 (0.37-1.59)	0.5	0.47 (0.13-1.72)	0.26	0.41 (0.12-1.5)	0.18	NC	NC	2.91 (0.18-46.36)	0.45
PD-L1 cutoff 1% (High vs Low)	0.41 (0.25-0.68)	*<0.001*	0.21 (0.07-0.64)	*0.01*	NC	NC	0.30 (0.04-2.2)	0.23	0.17 (0.03-0.89)	*0.04*
TMB cutoff 10 (High vs Low)	1.19 (0.67-2.10)	0.55	0.75 (0.14-3.40)	0.74	1.00 (0.37-2.7)	0.99	0.55 (0.09-3.2)	0.51	NC	NC
TP53 (mutation vs WT)	1.00 (0.61-1.63)	0.99	1.24 (0.41-3.75)	0.70	NC	NC	0.41 (0.07-2.4)	0.33	9.78 (1.22-78.11)	*0.03*
STK11 (mutation vs WT)	2.25 (0.87-5.83)	0.10	4.53 (1.05-19.47)	*0.04*	NC	NC	NC	NC	NC	NC
KEAP1 (mutation vs WT)	1.48 (0.63-3.45)	0.37	2.13 (0.49-9.27)	0.32	NC	NC	1.32 (0.08-22.8)	0.85	NC	NC

Note: Values are shown as number (%) unless indicated otherwise.

† steroid use to treat irAE during ICI therapy; $ antibiotic use during ICI therapy; PFS, progression-free survival; AGA, actionable genetic alteration; HR, hazard ratio; CI, confidence interval; TMB, tumor mutational burden. NC, not calculable;

<u>Table S4. PFS analysis of PD-L1 expression in patients with AGA mutations (*EGFR*, *KRAS*, *HER2*, and *MET subgroups*).</u>

Survival Analysis		AGA n=324			KRAS n=72			EGFR n=149			HER2 n=34			MET n=32		
		PFS			PFS			PFS			PFS			PFS		
		n (%)	median (95%CI)	p	n (%)	median (95%CI)	p	n (%)	median (95%CI)	p	n (%)	median (95%CI)	p	n (%)	median (95%CI)	p
Clinical factor		324			72			149			34			32		
PD-L1 expression		T			72			145			33			31		
cutoff 1%	H (≥1%)	205 (64.7)	2.0 (2.0-3.0)	*<0.001*	46 (63.9)	4.0 (2.1-6.1)	*<0.001*	93 (64.1)	2.0 (2.0-2.0)	0.2	14 (42.4)	2.0 (2.0-NR)	0.1	23 (74.2)	5.1 (2.0-NR)	0.2
	L (<1%)	112 (35.3)	2.0 (2.0-2.0)		26 (36.1)	2.0 (1.0-3.0)		52 (35.9)	2.0 (2.0-2.0)		19 (57.6)	2.0 (1.0-3.0)		8 (25.8)	2.5 (1.0-NR)	

Note: Values are shown as number (%) unless indicated otherwise.

PFS, progression free survival; AGA, actionable genetic alteration; CI, confidence interval. H, High; L, Low NR, not reached.

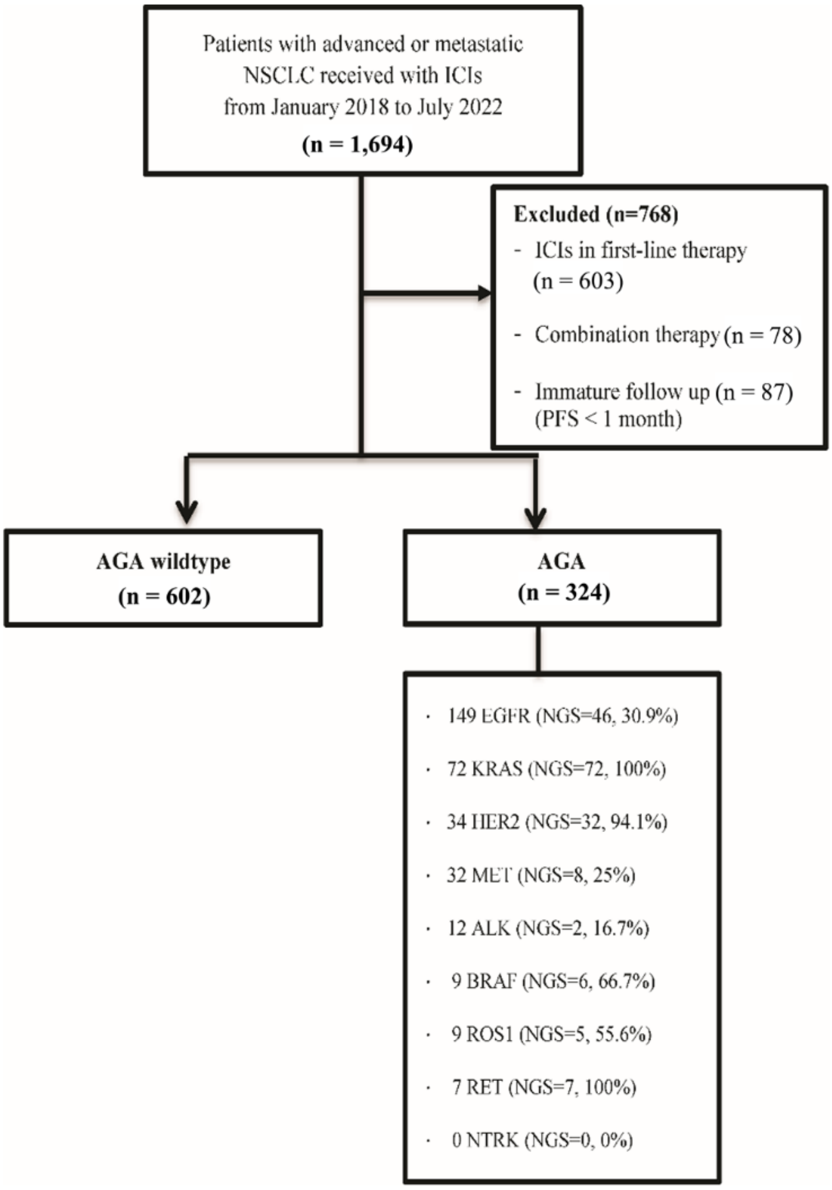

Figure S1. Patient flow diagram

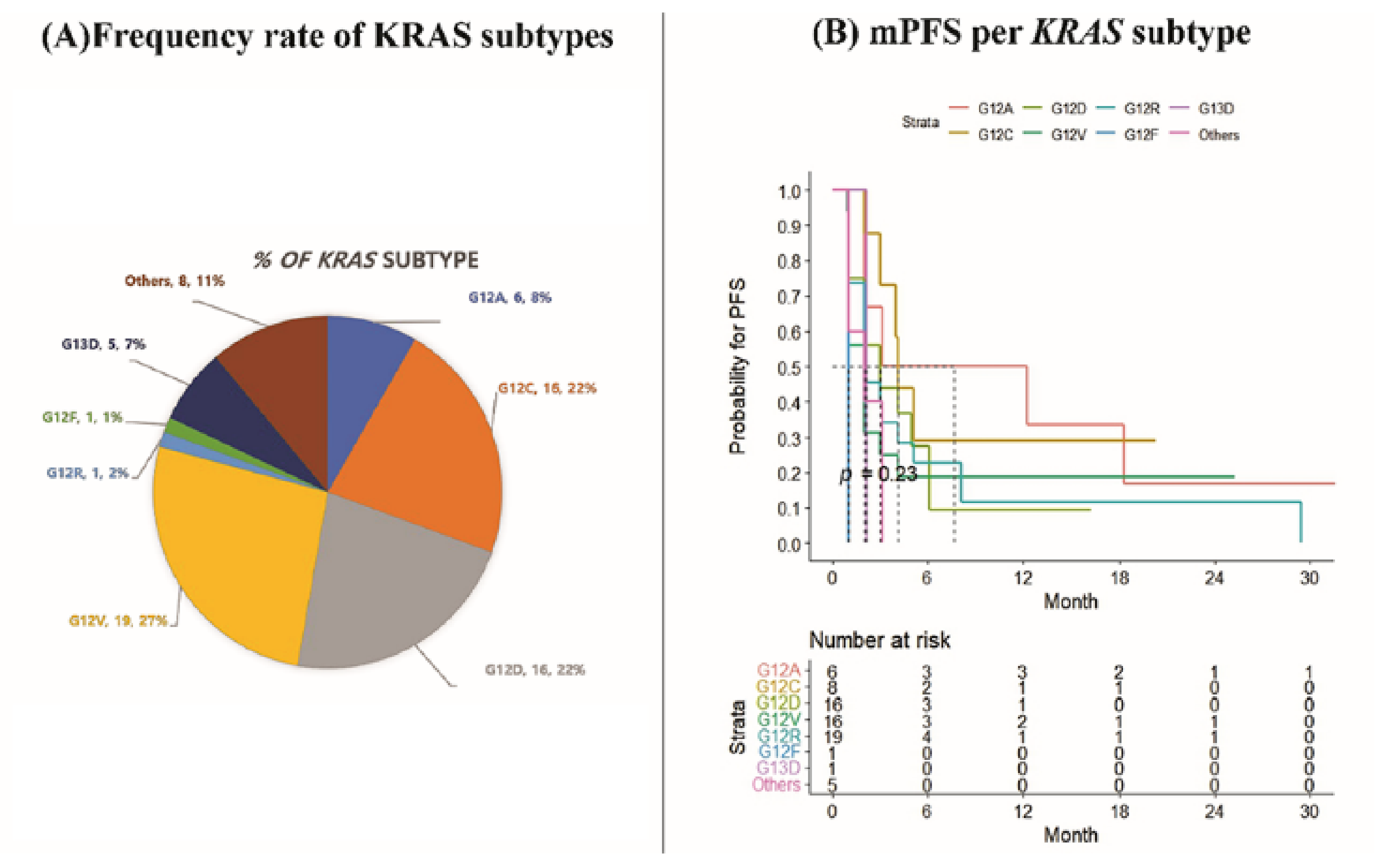

Figure S2. *KRAS* subtypes (A) Frequency rate of *KRAS* subtypes (B) mPFS per *KRAS* subtype.

PFS, progression-free survival
